# Hospital admissions in Austria during the COVID-19 Pandemic – a rapid analysis for 2020 and 2021

**DOI:** 10.1093/eurpub/ckac129.167

**Published:** 2022-10-25

**Authors:** H Ostermann, K Eglau

**Affiliations:** 1 Austrian National Public Health Institute, Gesundheit Österreich GmbH, Vienna, Austria; 2 Department of Public Health and HTA, University for Health Sciences, Medical Informatics and Technology, Hall/Tyrol, Austria

## Abstract

**Background:**

The COVID-19 pandemic posed a substantial shock to health service provision, in particular regarding hospital services. The reasons and also rationales for reduced health service provision were manifold ranging from limited supply due to resource restrictions, limited demand in order to avoid infections or due to reduced incidence of various diseases, and postponement of elective services. Nevertheless, the provision of services for acute care at an adequate level is paramount to avoid patient harm.

**Methods:**

Hospital admissions were analysed via administrative DRG data reported by Austrian hospitals. We compared health service provision on a monthly basis between 01/2018 and 12/2021. Services were classified according to ICD-10 and encompassed admissions due to acute heart failure, stroke, accidents, knee and hip surgery and breast cancer surgery.

**Results:**

Our findings show that hospital admissions for acute heart failure decreased by up to 25% between 03/2020 and 05/2020. In contrast, no significant difference to the initial pre-pandemic levels could be observed in the later stages of the pandemic. Stroke admissions remained at the initial levels throughout the whole period of analysis, while a substantial decrease (up to 50%) in admissions because of accidents was observed whenever severe NPIs were in place. Knee and hip surgery levels dropped in line with increasing ICU occupancy rates caused by COVID-19 patients. Decreases in breast cancer surgery could only be observed (up to 20%) during the four months of the pandemic (03-06/2020).

**Conclusions:**

Our analysis provides an aggregated insight into service provision management in Austrian hospitals throughout the pandemic. While acute care was continuously provided for most areas of diseases and elective surgeries were widely postponed in line with pressure on ICU capacities, the decline in breast cancer surgery demands attention and further clarification of whether this decline was supply- or demand-driven.

**Key messages:**

• During the pandemic inpatient acute care services were continuously provided for most diseases in Austrian hospitals while elective surgeries were postponed in line with pressure on ICU capacities.

• The pandemic posed a substantial challenge to service provision management in hospitals and unwarranted levels of service provision so far indicate areas of action for future (pandemic) preparedness.

